# Quantifying the use of connected digital products in clinical research

**DOI:** 10.1038/s41746-020-0259-x

**Published:** 2020-04-03

**Authors:** Caroline Marra, Jacqueline L. Chen, Andrea Coravos, Ariel D. Stern

**Affiliations:** 1 Harvard Business School, Boston, MA USA; 2 Harvard-MIT Center for Regulatory Science, Boston, MA USA; 3 Elektra Labs, Boston, MA USA; 4 Digital Medicine Society (DiMe), Boston, MA USA

**Keywords:** Clinical trials, Outcomes research

## Abstract

Over recent years, the adoption of connected technologies has grown dramatically, with potential for improving health care delivery, research, and patient experience. Yet, little has been documented about the prevalence and use of connected digital products (e.g., products that capture physiological and behavioral metrics) in formal clinical research. Using 18 years of data from ClinicalTrials.gov, we document substantial growth in the use of connected digital products in clinical trials (~34% CAGR) and show that these products have been used across all phases of research and by a diverse group of trial sponsors. We identify four distinct use cases for how such connected products have been integrated within clinical trial design and suggest implications for various stakeholders engaging in clinical research.

## Introduction

Over the past decade, connected technology has flooded health product markets with more than 116 million wearables shipped globally in 2018, and forecasts suggest that sales will double over the next 5 years (https://www.businesswire.com/news/home/20190516005571/en/World-Market-Connected-Wearables-4th-Edition-Shipments). Though many connected digital products have been primarily designed for nonclinical consumer use, clinicians and researchers can also use these products to capture real-time behavioral and physiological data from patients outside the clinic (e.g., heart rate, step counts, and sleep patterns). The use of digital products to remotely monitor patients in clinical research is not new; in fact, some early digital measurement products, such as Holter monitors and continuous blood glucose meters, have been used in clinical trials for decades. Yet, there is a dearth of data on how often and in what context such products have been integrated into clinical research—information that has implications for stakeholders across the research ecosystem.

**Fig. 1:**
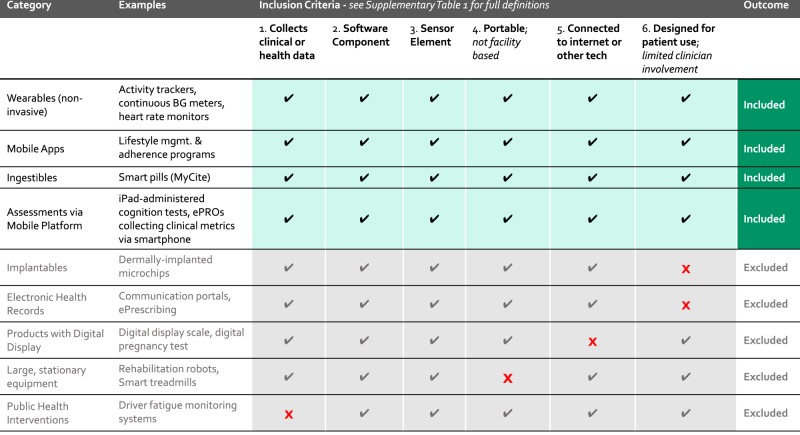
Defining “connected digital products”—inclusion criteria. Products must meet all six of the criteria labeled along the top of this figure in order to be included in this study as “connected digital products”. Products that fail to meet any one of the six criteria are excluded.

In this study, we document the use of “connected digital products,” a term which encompasses innovative technologies that are software driven, sensor based, and patient focused—elsewhere in the literature these and similar products may be referred to as wireless and digital assessment tools. Specifically, all of the products included in our study meet six criteria: the product must (1) collect *clinical or health-related* measurements, (2) include a *software* component^1^, (3) include a *sensor* element, such as bio measurement or touch sensor, (4) be *portable* so that the patient does not need to go to a facility to use the product, (5) have the capability to *connect* to the internet or another device (e.g., via Bluetooth, mobile app, USB), and (6) be designed for *patient use* with little to no clinician involvement required (Fig. 1). See Supplementary Table 1 for full definition.

To identify connected digital products that meet this definition, we created a comprehensive list of product model names and manufacturers (derived from four existing sources) and searched for these terms in records downloaded from ClinicalTrials.gov using a comprehensive text search algorithm. We also included several general terms without commercial identifiers to capture trials where the product name or manufacturer was not explicitly mentioned (e.g., “smart watch”).

An emerging body of literature illustrates that connected digital products are playing a growing role in clinical research. A few recently published papers have used online literature sources (e.g., Pubmed, Embase, and Medline) to track how frequently medical researchers are incorporating mobile and remote monitoring technologies into their clinical studies^2–4^. One study also used ClinicalTrials.gov to document how internet-connected technology is being studied in cancer-specific applications^5^. Other research-focused organizations have made efforts to track clinical research involving the use of a particular brand of wearable product (https://www.fitabase.com/). Our study extends existing work in this emerging field by considering the entire landscape of clinical research across disease areas and study types, aiming to quantify the rate of adoption of connected digital products in registered clinical trials. Furthermore, we provide detail as to the various ways trial sponsors are using digital products in clinical trials.

## Results

**Fig. 2:**
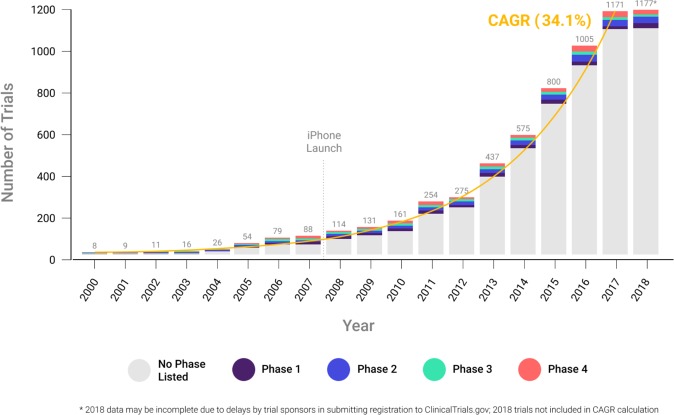
Clinical trials using connected digital products by study start year and phase. Each bar represents the total number of clinical trials started annually that include a connected digital product. The trials are segmented by phase, as designated in the ClinicalTrials.gov record.

Our analyses document substantial growth in the use of connected digital products in clinical trials in the years since 2000 with a compound annual growth rate of ~34%. Notably, in both 2017 and 2018—the most recent years in our sample—over 1100 unique trials included use of a connected digital product, a more than tenfold increase over the same count in the early 2000’s (Fig. 2).

Furthermore, we find that connected digital products have been used across all phases of clinical research. Approximately 13% of trials using connected digital products are designated as either FDA development stage trials (phase 1, 2, 3) or post-marketing studies (phase 4) and the distribution across phase 1–4 trials is relatively equal, suggesting the utility of digital products across multiple phases of clinical research. The remainder of the trials were not formally linked to an FDA-defined phase; these appear to be evidence-generating studies, in which the sponsor can include a variety of exploratory or confirmatory objectives, suggesting growing interest in effectively and safely deploying connected digital products in clinical research.

In addition, both industry (e.g., pharmaceutical and medical device firms) and nonindustry (e.g., government and nonprofit) organizations have been involved in sponsoring trials that use connected digital products. Since 2000, ~19% of trials using a connected digital product (nearly 1200 trials total) have included an industry sponsor or collaborator.

**Fig. 3:**
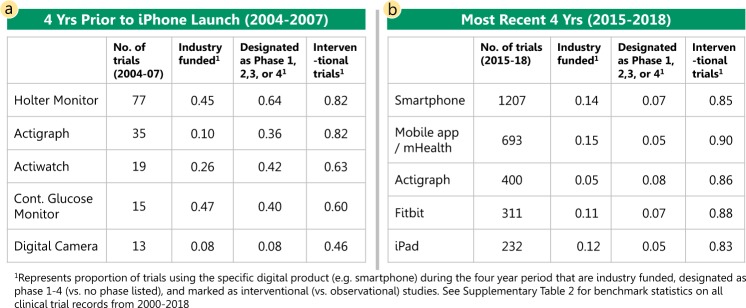
Most frequently used connected digital products. **a** The most commonly used connected digital products during the 4 years prior to the iPhone launch are reported with statistics describing the number of trials and key characteristics of the trials for each product during the period 2004–2007. **b** Similarly, the most commonly used connected digital products during the most recent four years of our data, 2015–2018, are described.

Unsurprisingly, the types of connected digital products most frequently used in registered trials has evolved over time. In the years leading up to the introduction of the iPhone (2004–2007), Holter monitors, activity watches, and continuous blood glucose monitors were the primary types of connected digital products used in trials, whereas, in the most recent years (2015–2018) smartphone-enabled technology and mobile applications have become the most commonly used types of connected digital products (Fig. 3). The mix of trial sponsors also appears to have shifted over time to include more trials led by nonindustry funders. See Supplementary Table 2 for benchmarks from all ClinicalTrials.gov records.

**Fig. 4:**
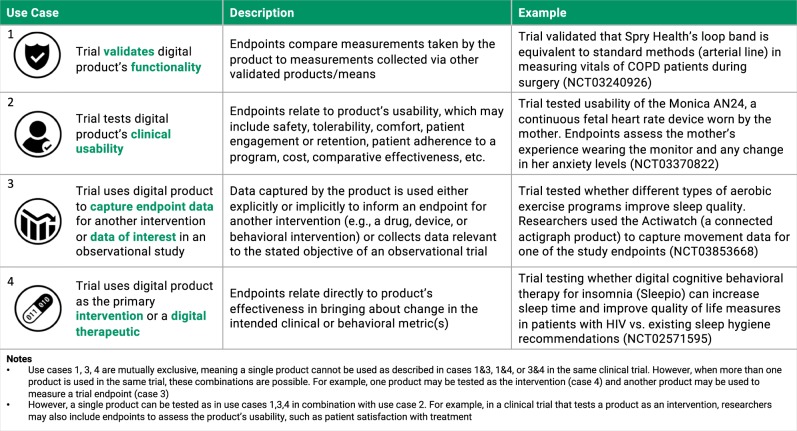
Classifying connected digital product use in clinical trials. Connected digital products are used in clinical trials in four unique ways. Each method of use is described and an example trial from this study’s dataset is included.

Notably, the ClinicalTrials.gov data point to several different ways in which investigators are incorporating connected digital products into their clinical studies. In reviewing the trials, we identified four different use cases. Generally, a trial can be designed to (1) verify or validate the digital product, (2) test the digital product’s clinical usability, (3) deploy the product to collect endpoint data to measure the impact of an intervention, or (4) use the product as the intervention itself (e.g., digital therapeutic). Figure 4 presents greater detail around these use cases alongside examples of trials from this study’s dataset that are characteristic of each approach.

## Discussion

The recent growth in the use of connected digital products in clinical trials (~34% CAGR over the period beginning in 2000 through 2017) suggests a number of opportunities for the future of clinical research. By enabling remote patient monitoring and enhancing the quality, quantity, and frequency of data collection, connected digital products introduce new opportunities to improve the efficiency of clinical trials. For example, trials that include connected digital products can be powered by more robust and continuous data measurement, potentially decreasing the need for large sample sizes of individual participants and increasing the speed at which adaptive trial decisions can be made^6^. The use of these products also allows for inclusion of novel trial endpoints and biomarkers that are digitally collected (https://www.statnews.com/2019/11/06/digital-endpoints-library-clinical-trials-drug-development/ and https://www.ctti-clinicaltrials.org/files/novelendpoints-recs.pdf). Furthermore, connected digital products that collect real-world evidence can enable novel trial formats like decentralized clinical trials—i.e., studies conducted outside of the physical boundaries of the clinic^7^.

Our findings suggest that researchers are beginning to discover new ways to use connected digital products to advance the execution of clinical studies. Though connected digital products are found in trials across FDA-defined phases, the large share without a specified phase appear to be evidence-generating studies. The focus on this type of exploratory research suggests that trial sponsors from both industry and nonindustry settings are experimenting with a variety of novel ways to include data collected by connected digital products in their research.

The increasing use of connected digital products in clinical trials has important implications for stakeholders across the research ecosystem. For product developers, verification and validation of digital products through clinical studies is a necessary first step to stimulate broader adoption in the clinical research and care settings^8^. For pharmaceutical manufacturers and contract research organizations, the emergence of connected digital products into the clinical research space presents an opportunity to include novel trial endpoints that use real-world evidence. Finally, for patients, connected digital products can reduce the burden of trial participation and increase the inclusivity of clinical research by fostering remote monitoring and encouraging the enrollment of individuals who might otherwise be unable to participate due to socioeconomic circumstances or travel limitations.

Because ClinicalTrials.gov does not require trial sponsors to disclose whether the data measurement tools used in the trial are connected digital products (they are only required to disclose the type of measurement, like FEV1 or HbA1c), we cannot be certain that all trials using a connected digital product were captured in our dataset. Furthermore, though we generated over 1000 search terms from the model and manufacturer names (using product lists from four existing sources) and identified a set of general terms to capture trials where commercial identifiers were not referenced, our list of search terms may be incomplete. Conversely, while we have done extensive manual validation of the search terms and clinical trials in our dataset by reading and evaluating a diverse subset of trials across years, stage of research, and funder types, there may still be cases where trials that do not include a connected digital product were inadvertently included. Finally, due to variations in data entry by trial sponsors, we were unable to segment trials reliably based on other characteristics of interest, such as disease area. See Supplementary Table 3 for a summary of diseases and conditions addressed by 2018 trials as determined by the research team through manual review of 1 full year of trial records.

The work discussed here is part of a broader stream of research aimed at understanding the digital transformation of clinical research in the modern era. Though descriptive in nature, our findings document a noteworthy trend: the use of connected digital products in clinical trials has grown dramatically since 2000, at a compound annual growth rate of ~34%, and more than 1100 trials using these products were initiated in both 2017 and 2018. Furthermore, connected digital products are being used across all stages of trials, with support from a wide variety of sponsoring organizations.

To achieve broad adoption in clinical practice, verification and validation of connected digital products will be essential and, like other interventions, ongoing vigilance regarding their safety, efficacy, and usability will be valuable for clinicians and regulators. Future analysis in this area should explore in more detail the context in which different organizations are using connected digital products to bolster their research efforts.

## Methods

To identify connected digital products, we collated product lists sourced from the Atlas by Elektra Labs (https://elektralabs.com/digital-measures-atlas), CTTI Mobile Technologies Database (https://feasibility-studies.ctti-clinicaltrials.org/), Frost and Sullivan’s 2016 Wearable Technologies Report (https://store.frost.com/wearable-technologies-in-clinical-and-consumer-health-forecast-to-2020.html), and Scripps Research Digital Health Library (https://digitalhealthlibrary.scripps.edu/) to extract a comprehensive list of products’ model names and manufacturers. We also downloaded the complete set of clinical trial records available from the ClinicalTrials.gov database. ClinicalTrials.gov is a publicly-available resource provided by the United States (US) National Library of Medicine and includes over 312,000 research studies in the US and abroad. Since September 2007, the party or parties responsible for a clinical trial have been required to register on ClinicalTrials.gov when that trial is being used to support the regulatory approval of a new therapeutic product (e.g., a drug or medical device), and the International Committee of Medical Journal Editors requires ex ante trial registration in order to publish studies in any of its member journals (https://www.govinfo.gov/content/pkg/PLAW-110publ85/pdf/PLAW-110publ85.pdf#page=82 and http://www.icmje.org/recommendations/browse/publishing-and-editorial-issues/clinical-trial-registration.html). We limited the sample of clinical trials to those launched from 2000 to 2018, inclusive, where the current trial status showed that the trial had at least begun to recruit participants. See Supplementary Table 4 for full list of trial status categories from ClinicalTrials.gov.

To identify trials that incorporated connected digital products, we created a search term(s) for each product that was derived from either the product model name, the manufacturer, or a combination of the two. We also included several general search terms, such as “smartphone”, “actigraph”, “smart watch”, and so on, to capture trials where a certain type of connected digital product was used but not referenced by its model or manufacturer name. Since clinical trial information is entered manually by trial sponsors, the research team read a subset of trials to identify how investigators most commonly referred to products. See Supplementary Methods for examples of how search terms were derived and validated.

Using the comprehensive list of search terms generated, we performed an automated text search within the downloaded records from ClinicalTrials.gov. We used a comprehensive search algorithm, which allowed us to capture text in all relevant database fields where use of a digital product might be recorded. These included *outcome measures*, *intervention*, and *study description*, among others. See Supplementary Table 5 for full list of database fields searched. The research team also manually reviewed a random sample of trials from each year to ensure the search terms correctly identified relevant trials in which a connected digital product was actually used.

### Reporting summary

Further information on research design is available in the Nature Research Reporting Summary linked to this article.

## Supplementary information

Supplementary Information

Reporting Summary

## Data Availability

The data that support the findings of this study are publicly available at https://github.com/arieldora/ConnectedDigitalTools.

## Code availability

The code used to pull clinical trial data for this study is also available at https://github.com/arieldora/ConnectedDigitalTools.
