# Supplementary material for: Quantifying the use of connected digital products in clinical research
Source: NPJ Digit Med. 2020 Apr 3;3:50. doi: 10.1038/s41746-020-0259-x (PMC7125096; doi:10.1038/s41746-020-0259-x)
Supplement: Supplementary file 1 — Supplementary Information [file 41746_2020_259_MOESM1_ESM.pdf]

SUPPLEMENTARY MATERIALS

Supplementary Table 1:
Detailed Definition of "Connected Digital Products"

	**Connected Digital Products defined as meeting all of the following criteria:**
**1**	Collects clinical and health-related measurements, which is defined as measuring biomarkers and other health data useful for clinical trials.
**2**	Includes software component, which is broadly defined to include both software and algorithms. For more detail, see Coravos, Khozin, Mandl (2019)
**3**	Includes sensor component, broadly defined, that can be a bio-sensor or touch sensor (e.g., measurement of keyboard strokes, touchpad entry)
**4**	Portable meaning that the patient/consumer does not need to go to a specific facility or location to use the product
**5**	Connected to internet or other technology where the connection may be facilitated by, e.g., Bluetooth, mobile app, USB.
**6**	Designed for patient use meaning that the product is intended to be used and/or operated by the patient/consumer with little-to-no clinician involvement required.

Supplementary Table 2:
Clinical Trial Status Designations in ClinicalTrials.gov Records
Trials with all statuses except those in gray were included in this study.

**Trial Status**	**Description from ClinicalTrials.gov**
Not yet recruiting	Study has not started recruiting participants
Recruiting	Study is currently recruiting participants
Enrolling by invitation	Study is selecting its participants from a population, or group of people, decided on by the researchers in advance
Active, not recruiting	Study is ongoing, and participants are receiving an intervention or being examined, but potential participants are not currently being recruited
Suspended	Study stopped early but may start again
Terminated	Study stopped early and will not start again. Participants are no longer being examined or treated.
Completed	Study ended normally, and participants are no longer being examined or treated (that is, the last participant's last visit has occurred)
Withdrawn	Study stopped early, before enrolling its first participant
Unknown	Study whose last known status was recruiting; not yet recruiting; or active, not recruiting but that has passed its completion date, and status has not been last verified within the past 2 years

Supplementary Table 3:
Full List of ClinicalTrials.gov Database Fields Searched for Connected Digital Products

Database Fields Included
intervention/treatment
intervention/treatment description
outcome measures
outcome measures description
study arm
study arm description
brief summary
official title
detailed description

Supplementary Table 4:
ClinicalTrials.gov Records – Benchmark Statistics
The following includes benchmark data from all clinical trial records found in the ClinicalTrials.gov database with a study start date from 1/1/2000 – 12/31/2018 (regardless of whether or not a connected digital product was used). Note: data was pulled Nov. 30, 2019 and is subject to change as trial sponsors may retrospectively add new trials and modify existing records.

	Clinical Trials (2000-2018)
Total Records	282,879
Industry Funder	34% (of total records)
FDA-defined Phase	52% (of total records)<br>[14% phase 1; 18% phase 2; 11% phase 3; 9% phase 4]
Interventional Study	79% (of total records)

Supplementary Table 5:
Primary Conditions Addressed by Trials using Connected Digital Products (2018 Study Start Dates)
The primary medical and/or health condition addressed in the clinical trial was assessed manually by two researchers reading in duplicate for trials using connected digital products that were started in 2018. This data is presented for illustrative purposes and is only representative of the conditions and disease areas where connected digital products have most recently been used in clinical trials.

Primary Condition	No. Trials Started in 2018
Physical Activity/Weight Management	223
Mental Health	145
Neurological/Musculoskeletal	88
Rehabilitation	73

Endocrine	68
Cardiovascular	66
Sleep	60
Pain	41
Care Coordination	39
Women's & Sexual Health	30
Respiratory	28
Cancer	22
Medication Adherence	16
Bowel/Kidney	15
Ophthalmology	12
Primary Care	11
Vital Signs Monitoring	11
Other	27

Notes: A few trials that started in 2018 were excluded during manual review for various reasons; e.g., digital product was not used in a manner that met the definitional criteria or information provided by trial sponsor was insufficient. Only trials registered on CliicalTrials.gov as of July 2019 were included in the research team's manual review, so trials that started in 2018 but were not registered in the public database until after July 2019 are not included in the above figure.

Supplementary Methods:
Detailed Process for Deriving Search Terms from Product Model and Manufacturer

<u>Step 1:</u>
In order to identify trials that incorporated connected digital products, we started with the consolidated list of product make and model names (the consolidated list was collated by the research team from four existing sources). We created a search term(s) for each product that was derived from either the product model, the manufacturer, or a combination of the two. Since clinical trial information is entered manually by trial sponsors, the research team input a variety of potential search terms into the ClinicalTrials.gov database and then read a subset of the trials identified for each product to determine how investigators most commonly referred to the product (e.g., model name only) and to ensure the search term would return only trials that actually used a connected digital product and not erroneous results.
For example:

- If the model name was distinct and easily referenceable, it was used as the search term. For example, "activPAL" was the search term for the activPAL$^{(R)}$ product made by PAL technologies. To determine this, the research team tested several combinations of the product make and model name, such as "PAL technologies", "activPAL", "PAL activPAL", and "PAL technologies activPAL" but found in a review of the ClinicalTrials.gov records that trial sponsors consistently referred to the product as simply "activPAL" In a couple of trials, the trial sponsors mistakenly referred to the product as "activePAL" so this alternate spelling was also included as a search term for the product.

- If the model name was not distinct and could be confused with other references, both the model and manufacturer names were included as a string-based search term. For example, the UP devices originally manufactured by the company Jawbone could not be searched by “up” or “jawbone” alone because in testing potential search terms for this product, the research team found that both words appear in the text of clinical trial descriptions other contexts (e.g. jawbone within the mouth and the motion “up"). Therefore, the text string “Jawbone UP” was used as the search term.
- In some cases, the model name was not referenced in clinical trials, so the manufacturer name was used. This was especially relevant when the company was well-known and made several (exclusively) digital products, for example, the research team found that “Garmin” was the most appropriate search term for all Garmin smartwatches and chestbands as trial sponsors often use variants of the product name itself but always seem to include the word Garmin (e.g. Garmin watch, Garmin band, chestband made by Garmin)

Step 2:

After creating search terms from our consolidated list of products with commercial identifiers, the research team tested several general search terms (e.g. “wearable”, “smartwatch”, “smart inhaler”). These terms are not linked to a particular branded product but are representative of broader product categories and are included to ensure the search captured those trials where connected digital products were used but the model or manufacturer name was not explicitly mentioned. The research team also read a subset of trials returned by the general search terms to ensure the terms selected for inclusion accurately identified trials that used a connected digital product.
